# Interleukin Variants Are Associated with the Development and Progression of IgA Nephropathy: A Candidate-Gene Association Study and Meta-Analysis

**DOI:** 10.3390/ijms242216347

**Published:** 2023-11-15

**Authors:** Ioanna Chronopoulou, Maria Tziastoudi, Georgios Pissas, Efthimios Dardiotis, Maria Dardioti, Spyridon Golfinopoulos, Georgios Filippidis, Peter R. Mertens, Evangelia E. Tsironi, Vassilios Liakopoulos, Theodoros Eleftheriadis, Ioannis Stefanidis

**Affiliations:** 1 Departments of Nephrology, Faculty of Medicine, School of Health Sciences, University of Thessaly, 41334 Larissa, Greece; ichron@yahoo.gr (I.C.); matziast@med.uth.gr (M.T.); gpissas@msn.com (G.P.); spygolfin@yahoo.gr (S.G.); gfilippid@yahoo.gr (G.F.); teleftheriadis@med.uth.gr (T.E.); 2 Laboratory of Neurogenetics, Department of Neurology, University of Thessaly, University Hospital of Larissa, 41334 Larissa, Greece; edar@uth.gr (E.D.); madardio@yahoo.gr (M.D.); 3 University Clinic for Nephrology and Hypertension, Diabetology and Endocrinology, Medical Faculty, Otto-von Guericke University Magdeburg, 39120 Magdeburg, Germany; peter.mertens@med.ovgu.de; 4 Department of Ophthalmology, Faculty of Medicine, School of Health Sciences, University of Thessaly, University Hospital of Larissa, 41334 Larissa, Greece; e_tsironi@hotmail.com; 5 2nd Department of Nephrology, AHEPA Hospital, School of Medicine, Aristotle University of Thessaloniki, 54636 Thessaloniki, Greece; vliak@auth.gr

**Keywords:** gene polymorphism, immunoglobulin A nephropathy, interleukin-1α, *IL1A*, interleukin-1β, *IL1B*, interleukin-1 receptor antagonist, *IL1RN*, *IL10*

## Abstract

The interleukin-1 gene cluster encodes cytokines, which modulate mesangial cell proliferation and matrix expansion, both constituting central factors in the development and progression of immunoglobulin A nephropathy (IgAN). A candidate-gene study was performed to examine the association of polymorphisms of the interleukin-1 gene cluster with the risk of progressive IgAN. To gain deeper insights into the involvement of interleukin genes in IgAN, a meta-analysis of genetic association studies (GAS) that examine the association between interleukin variants and IgAN was conducted. Association study: The case-control study consisted of 121 unrelated Caucasians with sporadic, histologically diagnosed IgAN and of 246 age- and sex-matched healthy controls. Persistent proteinuria (>2 g/24 h) and/or impaired kidney function (serum creatinine > 1.5 mg/dL) defined progressive (n = 67) vs. non-progressive (n = 54) IgAN cases. Genotypes were assessed for two promoter-region single-nucleotide polymorphisms, C-899T (rs1800587) in *IL1A* and C-511T (rs16944) in *IL1B*, and for one penta-allelic variable-length tandem repeat polymorphism (VNTR 86 bp intron 2) in *IL1RN*. The association of these variants with the susceptibility of IgAN and the development of progressive IgAN (healthy status, IgAN, progressive IgAN) was tested using the generalized odds ratio (OR_G_) metric. Linkage disequilibrium and haplotype analysis were also performed. Meta-analysis: We included in the meta-analysis 15 studies investigating association between 14 interleukin variants harbored in eight different genes and IgAN. The OR_G_ was used to evaluate the association between interleukin variants and IgAN using random effects models. The present case-control study revealed association of *IL1B* C-511T (rs16944) with the progression of IgAN (*p* = 0.041; OR_G_ = 2.11 (1.09–4.07)). On haplotype analysis, significant results were derived for the haplotypes C-C-1 (*p* = 0.005; OR = 0.456 (0.261~0.797)) and C-T-2 (*p* = 0.003; OR = 4.208 (1.545–11.50)). Regarding association and meta-analysis results, variants in *IL1B* (rs1143627 and rs16944), *IL1RN* (rs928940, rs439154, and rs315951) and *IL10* (rs1800871) were associated with IgAN based on either genotype or allele counts. Genetic variants and haplotypes in the *IL1B*, *IL1RN,* and *IL10* genes might contribute to an increased risk for development and progression of IgAN.

## 1. Introduction

Immunoglobulin A nephropathy (IgAN) is one of the leading causes of end-stage renal disease in developed countries [1,2]. The clinical presentation of IgAN can vary greatly, encompassing a wide spectrum of manifestations. These can range from no apparent symptoms, such as the presence of asymptomatic hematuria and proteinuria, to gross hematuria accompanied by a rapid decline in kidney function [3]. Renal biopsy is considered the gold standard for diagnosis of IgAN. The typical observations through immunofluorescence microscopy in IgAN primarily involve the presence of IgA deposits within the mesangial region. These deposits are frequently accompanied by IgM, IgG, or complement C3 components [4]. In the pathophysiology of IgAN a crucial role has been shown for inflammatory cytokines including interleukin-1 (IL-1), a potent mediator with a central role in the inflammatory cascade [5].

Interleukin-1 is involved in mesangial cell proliferation and extracellular matrix production in various glomerulopathies [6,7]. In cases with IgAN, there is significant glomerular interleukin-1 expression [8], which may be linked to mesangial cell activation [9] in response to nephritogenic immune complexes. Interleukin-1 may act as an autocrine growth factor, enhancing mesangial cell proliferation [10]. Moreover, experimental evidence suggests that in IgAN, interleukin 1 released by mesangial cells could potentially play a role in the development of tubular damage and interstitial fibrosis [11,12,13], both of which are closely associated with the progression of the disease [14]. There are findings that indicate an association between the *IL1B* and *IL1RN* genes and heightened vulnerability to IgAN in children [15]. Additionally, a connection between the emergence of proteinuria in IgAN and IL1A has been postulated, while IL1B is linked to podocyte foot process effacement [15]. IL-1β has been showed to participate in human and experimental human diseases [16,17,18]. The activity of interleukin-1 (IL-1) in the urine has been also observed to be elevated in individuals with IgAN and Henoch–Schönlein nephritis (HSN) compared to those in a healthy control group [19]. The urinary levels of IL-1ra in IgAN patients were also found to be lower when compared to both healthy controls and HSN patients [20]. This discovery suggests a potential distinction in the underlying causes of these two diseases. In addition, it has been shown that the pro-inflammatory cytokine IL-1 is elevated in individuals undergoing maintenance hemodialysis (MHD), a population characterized by persistent inflammation, and is linked to heightened mortality rates [21]. The introduction of IL-1ra to patients undergoing maintenance hemodialysis (MHD) has been reported to be able to decrease markers associated with inflammation [21].

Ιt is reasonable, therefore, to propose that the interleukin 1 gene cluster might have a role in the development and progression of IgA nephropathy. In the present candidate-gene study we determined the genotypes for two single-nucleotide polymorphisms, C-899T in the interleukin-1α gene (*IL1A*; rs1800587) and C-511T in the interleukin-1β gene (*IL1B*; rs16944), and for one penta-allelic polymorphism with variable numbers of an 86 bp tandem repeat (VNTR *1–*5) in intron 2 of the interleukin-1 receptor antagonist gene (*IL1RN*) in a homogeneous population of Caucasian origin (Greeks). We then estimated the association between these interleukin-1 gene cluster variants and the risk for development and progression of IgAN (from healthy status, to IgAN and, finally, to progressive IgA nephropathy). This association was calculated by the generalized odds ratio (OR_G_) as a genetic model-free approach [22,23]. We also conducted an analysis of haplotypes.

To further investigate the role of interleukins in the development and progression of IgA nephropathy, we searched in the literature for all available genetic association studies (GAS) which test the association between interleukin variants and IgAN and synthesized the results with meta-analysis.

## 2. Results

### 2.1. Association Analysis

#### 2.1.1. Clinical Profile of Participants

The study cohort consisted of 121 patients with histologically diagnosed IgAN and of 246 age- and sex-matched healthy controls. All participants were unrelated Caucasians of Greek origin. According to the predefined criteria of progressive IgA nephropathy, i.e., persistent proteinuria (>2 g/24 h) and/or impaired kidney function (serum creatinine > 1.5 mg/dL), 67 participants were categorized as progressive (cases) and 54 participants as non-progressive (diseased controls) IgA nephropathy.

Table 1 includes the demographic and clinical profiles of all participants. Among cases with progressive IgAN, 22.4% (n = 15) were patients with end-stage kidney disease (ESRD), 17.9% (n = 12) who were under chronic renal replacement therapy, and 4.5% (n = 3) with a kidney transplant. The distribution of age was as follows: 74.6% of progressive cases (n = 50), 87% of diseased controls (n = 47), and 84.6% of healthy controls (n = 208) were above 60 years old. In 51.2% of progressive cases and 51.9% of diseased controls, the duration of IgAN was more than 5 years.

Exclusion of patients with ESRD (n = 15) partially changed the clinical profile of cases with progressive IgAN. However, between these cases and the diseased controls there was still no significant difference in disease duration (5.2 ± 4.0 vs. 5.7 ± 4.3 years; *p* = 0.565). After exclusion of patients with ESRD, frequency of macroscopic hematuria was significantly lower in cases with progressive IgAN than in diseased controls (5.3 vs. 11.9%; OR = 0.239 (0.063–0.915)).

#### 2.1.2. Development of Progressive IgA Nephropathy

The genotype distribution of the three variants of the interleukin-1 gene cluster in cases (progressive IgA nephropathy), diseased controls (non-progressive IgA nephropathy), and healthy controls, as well as the respective OR_G_, are shown in Table 2. The healthy controls conformed to the HWE for all variants (*p* ≥ 0.05).

There was no significant association between genotype distribution of these variants in comparison of healthy controls versus diseased controls versus cases (Table 2) or in comparison of cases versus healthy controls (*p* ≥ 0.05) (Table 3). However, there was significant association between *IL-1B* C-511T (rs16944) polymorphism and disease progression (*p* = 0.041). The model-free approach (OR_G_) produced significant results for the *IL-1B* C-511T (rs16944) variant, indicating that the variant mutational load plays a significant role in the development of progressive IgAN (Table 4).

#### 2.1.3. Linkage Disequilibrium Analysis

Table 5 shows the results of the linkage disequilibrium (LD) test between pairs of the interleukin-1 gene cluster variants. All variant pairs were in LD (*p* < 0.05) in both populations (cases and diseased controls), except for the variants rs1800587 C-899T in *IL1A* and the penta-allelic variant VNTR (86 bp intron 2) in *IL1RN* in diseased controls.

#### 2.1.4. Analysis of Haplotypes

The distribution of the estimated haplotype frequencies of the three interleukin-1 gene cluster variants (rs1800587 C-899T in *IL1A*, rs16944 C-511T in *IL1B,* and VNTR 86 bp in *IL1RN*) for cases with progressive IgAN and diseased controls is summarized in Table 6. The overall difference between progressive cases and diseased controls is significant (*p* = 0.003). In the analysis of the individual haplotypes (rs1800587 C/T *IL1A*, rs16944 C/T *IL1B*, 86 bp VNTR 1–5 *IL1RN*), significant results were derived for the haplotypes C-C-1 (*p* = 0.005; OR = 0.456 (0.261~0.797)) and C-T-2 (*p* = 0.003; OR = 4.208 (1.545–11.50)). The haplotype C-C-1 may be protective for progressive IgAN, as the allele T of rs16944 C/T in *IL1B*, which was shown to increase the risk of progressive IgAN, is missing in the haplotype. In contrast, haplotype C-T-2, which includes the risk allele T in *IL1B*, may increase the risk of progressive IgAN.

AMOVA analysis showed that there was no subdivision between cases and controls (F*st* = 0.00).

### 2.2. Meta-Analysis

#### Study Characteristics

**Figure 1:**
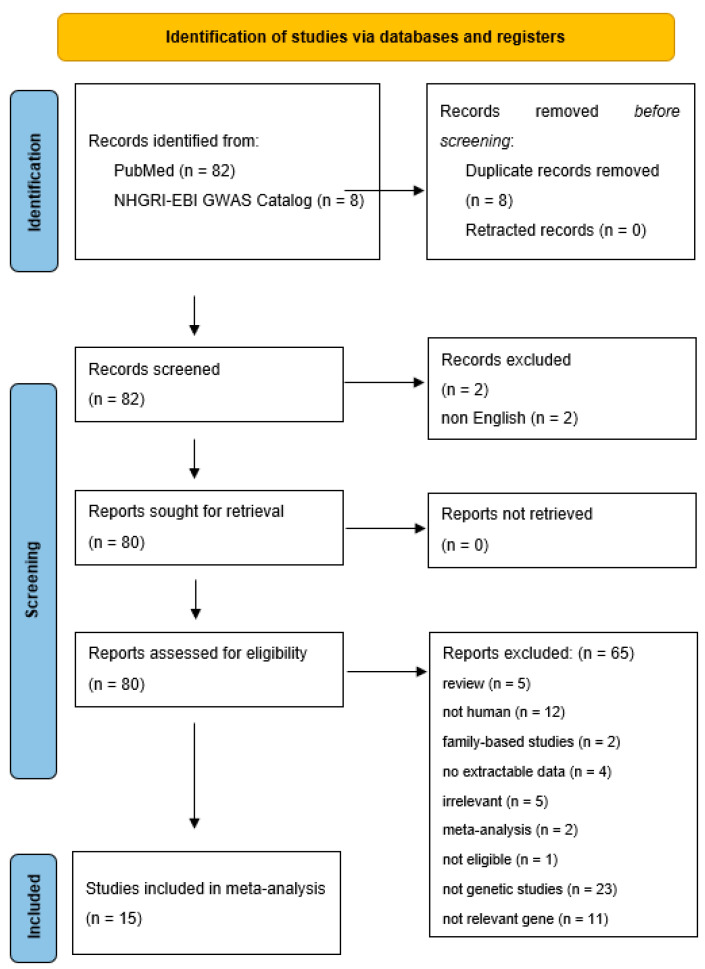
Flow chart showing how studies were selected for meta-analysis.

Research was carried out in diverse population groups with varying racial backgrounds, encompassing 11 studies involving Asian participants and 4 studies involving individuals of Caucasian descent. Figure 1 displays a flowchart illustrating the retrieved articles, along with those excluded and the reasons for their exclusion. Detailed information regarding the characteristics of each study included in the analysis can be found in Table 7.

Among the 8 different interleukin genes for which there are available data, 14 genetic variants were examined for association with IgAN. Table 8 shows the results of both meta-analyses and analyses of single studies based on genotype counts, whereas Table 9 shows the results of both meta-analyses and analyses of single studies based on allele counts.

Regarding analyses based on genotype counts, four variants provided significant results in analyses of single studies and one variant was revealed significant in meta-analysis. More specifically, C-819T (rs1800871) in *IL10* was significant in meta-analysis with a poοled OR_G_ of 0.79 (95% CI 0.64–0.97), whereas rs1143627 in *IL1B* (OR_G_ = 1.44 (95% CI 1.08–1.93)0 and rs928940, rs439154, and rs315951 in *IL1RN* were significant in analyses of single studies [(OR_G_ = 0.72 (95% CI 0.54–0.97), OR_G_ = 0.68 (95% CI 0.50–0.92), and OR_G_ = 0.73 (95% CI 0.55–0.98), respectively)] (Table 8). In analyses based on allele counts, significant association were reported for C-511T in *IL-1B* with a pooled OR_G_ of 1.35 (95% CI 1.05–1.75) (Table 9).

## 3. Discussion

The present study had two objectives. Firstly, it investigated whether certain variants of the interleukin-1 gene cluster, C-899T (rs1800587) in *IL1A*, C-511T (rs16944) in *IL1B*, and variable-length tandem repeat polymorphism (VNTR 86 bp 1–5) in *IL1RN*, are associated with the development and progression of IgAN, and afterwards, it synthesizes the available evidence about the involvement of interleukin variants in the development and progression of IgAN. According to the findings of our case-control study, C-511T (rs16944) in *IL1B* is implicated in progression of the disease. According to the findings of association and meta-analysis results, variants in *IL1B* (rs1143627 and rs16944), *IL1RN* (rs928940, rs439154, and rs315951) and *IL10* (rs1800871) were associated with IgAN based on either genotype or allele counts. The contribution of the *IL-1B* gene was also confirmed by another systematic review and meta-analysis, although the aforementioned study revealed significance for another variant (rs1143627) in the *IL-1B* gene [37].

Our study had several strengths. In examining the association between the above variants and progressive IgAN, we selected as a control group a population of diseased controls, i.e., subjects with non-progressive IgAN. This selection took place according to strict and well-defined criteria, i.e., persistent proteinuria (>2 g/24 h) and/or impaired kidney function (serum creatinine > 1.5 mg/dL), in order to clearly separate cases with progressive IgAN from diseased controls [38]. However, even IgAN with mild histological lesions—presenting with hematuria, only mild proteinuria, and normal renal function—might progress in about 30% of cases to severe kidney disease [39,40,41]. In this context, one third of the diseased control group (i.e., with non-progressive IgAN) is always a candidate to become a future case, i.e., a patient with progressive disease.

In order to categorize a given patient before inclusion in the study, laboratory data were collected on at least two separate occasions, three months apart from one another. However, in IgAN, a glomerulopathy with a characteristic chronic course, these data represent only a snap-shot of the disease status [42]. Finally, the key for a really strict categorization of patients with IgA nephropathy, having progressive or non-progressive disease, is a long-term follow-up.

In addition, the clinical practice in Europe includes renal biopsy only in patients with a more severe or progressive disease [38,42]. This fact means that the performance of a kidney biopsy is a priori favoring progressive disease and that histological diagnosis of IgAN is a serious selection bias. More specifically, every patient with IgAN who has undergone a renal biopsy potentially has progressive disease. Vice versa, a patient with microscopic hematuria, who has not undergone kidney biopsy, likely has a non-progressive disease. For these reasons, the diseased control group in our study certainly included only part of the whole spectrum of non-progressive IgAN. In the general population, the prevalence of mesangial IgA deposition and renal histology consistent with IgAN ranges from 3 to 16% [43,44].

The genotype distributions of the examined variants conformed to the HWE in the healthy control group, indicating lack of population stratification and genotyping mistakes [45]. The HWE asserts that, in the absence of disruptive factors, the genetic diversity within a population will remain stable across successive generations. Haplotype analyses revealed that two haplotypes are implicated in the susceptibility state “progressive” IgAN.

Another strength in our study was about the metric used to assess significance. We employed the generalized odds ratio (OR_G_) as a means to assess the magnitude of the association. This particular metric effectively addresses the challenge posed by multiple comparisons across various genetic models, including dominant, recessive, additive, co-dominant, and allele-contrast models. It accomplishes this by utilizing the complete genotypic data, thereby avoiding any ambiguity that may arise when multiple genetic models demonstrate significance. Consequently, the interpretation of the findings becomes more straightforward and robust. Furthermore, it eliminates the need for a predetermined selection of a specific genetic model. It is worth noting that this metric has been successfully applied in diverse research contexts, including studies related to conditions like diabetic nephropathy, IgAN, and chronic kidney disease [46,47,48,49,50,51,52].

However, our study also had some limitations. Despite finding some noteworthy associations, the sample size was relatively small, which is a common occurrence in candidate-gene association studies [53]. It is evident that a single institution cannot provide a sufficient number of patients to establish associations, especially if they truly exist. Therefore, future collaborative studies that pool data may offer more statistical power to detect significant associations [54]. Additionally, in the future, conducting meta-analyses of multiple studies could overcome the limitations of low statistical power and provide more conclusive evidence regarding the involvement of the interleukin 1 gene cluster in the progression of IgAN. Nevertheless, it is important to replicate the current findings in other gene-candidate or genome-wide association studies (GWAS) to validate their accuracy [55]. With the present meta-analyses, it is advisable to exercise caution when interpreting the findings, given the limited number of studies included in each meta-analysis. Last, but not least, applying the von Hippel correction for the I^2^ statistic due to the very small number of studies could offer a new perspective [56].

The progression of IgAN is a complex and multifactorial process influenced by epistatic and gene–environment interactions. Consequently, relying solely on single types of genetic studies, like gene-candidate association studies, may not yield definitive conclusions. To gain more conclusive evidence on the significance of the interleukin 1 gene cluster as predictors for progressive IgAN, a combination of hypothesis-driven and hypothesis-free studies is recommended. Hypothesis-driven studies, such as gene-candidate association studies, can be complemented by hypothesis-free studies like GWAS and microarrays gene expression analyses [57,58]. Integrating the findings from these different types of studies may provide a more comprehensive understanding of the disease’s genetic factors. GWAS, with their ability to unravel genetic complexity, hold promise in identifying significant genetic factors. However, replication of GWAS findings by different investigators and using different methodologies, including gene-candidate association studies, becomes crucial to interpreting the numerous associations that may arise from GWAS. While GWAS are valuable, gene-candidate association studies can support existing evidence and reveal genuine genetic effects that should be prioritized in future investigations. Thus, combining these different approaches can enhance our understanding of the genetic basis of progressive IgAN.

## 4. Materials and Methods

### 4.1. Association Study

#### 4.1.1. Participants

The study protocol was approved by the Ethics Committee of the University Hospital of Larissa, School of Medicine, University of Thessaly. All participants were recruited from patients at the University Hospital of Larissa, Greece. All patients attended the outpatient wards of nephrology between January 2019 and October 2019; only Caucasians of Greek origin were recruited after signing an informed-consent form.

The study cohort comprised three groups: cases with progressive IgAN, diseased controls (non-progressive IgAN), and healthy controls. Healthy controls were carefully matched to cases in terms of age and sex. Diagnosis was established based on histological findings from kidney biopsies [38,59]. The duration of IgAN was measured as the time elapsed (in years) from the date of kidney biopsy to the date of study inclusion. Progressive IgAN was defined by the presence of urinary protein excretion exceeding 2000 mg/24 h, indicative of overt proteinuria, with or without elevated serum creatinine levels (serum creatinine > 1.5 mg/dL). This definition was based on routine examinations conducted on at least two separate occasions, spaced three months apart, before the participant’s inclusion in the study. Infection was ruled out through prior urine dipstick testing. Additionally, the existence of arterial hypertension or cardiovascular disease, as well as recordings of arterial blood pressure (including systolic, diastolic, and mean arterial blood pressure), were noted for each participant [38]. Blood samples were collected from all individuals for biochemical measurements and DNA extraction.

#### 4.1.2. Genotyping

Genomic DNA was extracted from peripheral blood using a salting-out method. Genomic DNA was resuspended in 10 mM Tris-HCl and 1 mM ethylenediaminetetraacetic acid (EDTA), pH 8.0, and the concentration was measured by spectrophotometry. Enzymatic amplification of DNA was performed by polymerase chain reaction (PCR) and the genotyped variants were located on chromosome 2q14 on each one of the following interleukin-1 cluster genes: *IL1A* (spanning 112,773,915 to 112,785,394, overall 11,480 Kbp), *IL1B* (spanning 112,829,751 to 112,836,779, overall 7029 Kbp), and *IL1RN* (spanning 113,099,365 to 113,134,016, overall 35,652 Kbp).

The regions of the *Nco*1 polymorphic site at position −899 in the promoter region of the *IL1A,* of the *Ava*I polymorphic site at position −511 in the promoter region of *IL1B* and of the VNTR polymorphism within intron 2 of *IL1RN* were amplified according to previously described methods [60,61]. *IL1A* primers, sense 5′-TGTTCTACCACCTGAACTAGGC-3′ and antisense 5′-TTACATATGAGCCTTCCATG-3′, were used to amplify the PCR product including the C-889T polymorphism [61]. *IL1B* primers, sense 5′-TGGCATTGATCTGGTTCATC-3′ and antisense 5′-GTTTAGGAATCTTCCCACTT-3′, were used for the *IL1B* C-511T polymorphism [62].

The *IL1RN* primers, sense 5′-CTCAGCAACACTCCTAT-3′ and *IL1RN* antisense 5′-TCCTGGTCTGCAGGTAA-3′, were used to amplify the region within intron 2 of the *IL1RN* that encompasses the VNTR 86 bp polymorphism [60]. The PCR products were visualized by ethidium bromide staining on a 2% agarose gel. A band of 410 bp (four repeats of the 86 bp region) was classified as allele 1, a band of 240 bp (two repeats of 86 bp region) as allele 2, a band of 500 bp (five repeats of 86 bp region) as allele 3, a band of 325 bp (three repeats of 86 bp region) as allele 4, and a band of 595 bp (six repeats of 86 bp region) as allele 5 [60]. Alleles were categorized as L (including the long alleles 1, 3, 4, and 5) and 2 (the shorter allele 2). The respective genotypes were *IL1RN* L/L (long alleles), *IL1RN* L/2 (heterozygotes), and *IL1RN* 2/2 (allele 2 homozygotes).

#### 4.1.3. Data Analysis

Continuous variables were represented using mean values and standard deviations (mean ± SD), while categorical variables were presented as counts (or ratios) and percentages (n (%)). The normality of continuous variables was assessed using the Kolmogorov–Smirnov test. Pair-wise comparisons of continuous variables were conducted using the *t*-test or the Mann–Whitney U test for unpaired data, depending on the appropriateness. The frequencies of categorical variables were compared using either the χ^2^ test or Fisher’s exact test.

The study investigated the relationship between genotype distribution and disease progression, specifically the progression to IgA nephropathy, using the generalized linear odds ratio (OR_G_) [22,23]. The OR_G_ measures the likelihood of a subject being more diseased compared to less diseased, based on the higher mutational load in more diseased subjects relative to less diseased subjects [22,23]. Furthermore, the association between genotype distribution and disease status, which included healthy controls, diseased controls, and cases, was assessed using the χ^2^ test.

For healthy controls, the researchers evaluated the deviation of genotype distribution from the Hardy–Weinberg equilibrium (HWE) and checked for linkage disequilibrium (LD) between polymorphisms using exact tests following the approach by Weir [37,38]. A result was considered statistically significant when *p* < 0.05.

The examination of the HWE and LD was conducted using the Genetic Data Analysis (GDA 1.1) software [39,40], while haplotype frequencies were estimated and compared using SHEsis [63,64]. The OR_G_ was calculated with the help of ORGGASMA (https://biomath.med.uth.gr/default.aspx?lang=el&id=232164AC-9C6B-4A27-A595-2A22C35B6260&rid=576AB0F4-10AE-4BEA-8D97-C52B8B6BD4DA, accessed on 12 November 2023) [22,23]. GeneAIEX (v6.5) software was used to perform analysis of molecular variance (AMOVA) [65,66].

### 4.2. Meta-Analysis

#### 4.2.1. Identification and Eligibility of Relevant Studies

In order to clarify the role of interleukins in the development and progression of IgA nephropathy, we synthesized the results of all available GAS that examine the association between variants located in interleukin genes and IgAN. The studies were retrieved after extensive search of PubMed using the search terms ((“immunoglobulin A nephropathy” or “IgA nephropathy”) AND interleukin AND (gene OR polymorphism), accessed on 30 May 2023).

The collected publications underwent a comprehensive review to assess their suitability. Additionally, all references from the eligible studies were examined to identify any articles not included in the previously mentioned databases. Abstracts, case reports, editorials, review articles, in vitro studies, and family-based studies were excluded from the analysis. It is essential to mention that the search was limited to articles published in English. The eligibility of the articles was evaluated by two investigators, M.T. and I.S., and any differences in their assessments were resolved through mutual agreement.

The association studies included in the meta-analysis focused on the progression of IgAN. Both cases and controls consisted of patients with sporadic IgAN. Specifically, patients with a progressive form of IgAN were categorized as cases (progressors), while those with a stable nephropathy were categorized as controls (non-progressors). It is important to note that the eligibility criteria did not depend on a predefined definition of IgAN progression. Instead, each study’s own definition of progression was accepted and is presented in Table 7. Participants with other types of IgA nephropathy, such as Henoch–Schönlein purpura, as well as subjects with secondary IgA nephropathy, were excluded.

#### 4.2.2. Data Extraction

The first author, publication year, racial background of the study participants, selection criteria, demographic information, and complete genotype counts or allele counts were extracted from each study.

#### 4.2.3. Data Synthesis and Analysis

To explore the association between genotype distribution and the likelihood of developing sporadic IgAN or the risk of disease progression, we utilized the generalized linear odds ratio (OR_G_) [22,23]. When there were at least two studies available, a meta-analysis was performed, and the pooled odds ratio (OR) was calculated using random effects models (DerSimonian and Laird) [67]. All associations were reported as odds ratios (ORs) with corresponding 95% confidence intervals (CIs). To assess the heterogeneity among the studies, the Q-statistic was employed [68], and the degree of heterogeneity was quantified using the I^2^ metric [69].

For the analysis of the control group’s genotype distribution, the Fisher’s exact test was applied to test for deviation from the Hardy–Weinberg equilibrium (HWE). Additionally, the Egger test was used to examine small-study effects [70].

To conduct the generalized odds ratio methodology, the researchers utilized the ORGGASMA software (https://biomath.med.uth.gr/default.aspx?lang=el&id=232164AC-9C6B-4A27-A595-2A22C35B6260&rid=576AB0F4-10AE-4BEA-8D97-C52B8B6BD4DA, accessed on 12 November 2023) [22,23]. For all analyses, the Comprehensive Meta Analysis software package (CMA version 2; http://www.meta-analysis.com (accessed on 12 November 2023); 2005) and StatsDirect software 2013 (StatsDirect Ltd., Birkenhead, UK). StatsDirect statistical software 2008. http://www.statsdirect.com (accessed on 12 November 2023). StatsDirect Ltd.: Birkenhead, UK were employed.

## 5. Conclusions

In conclusion, the present study showed that genetic variations within the interleukin-1 gene cluster may contribute to an increased risk for “progressive” disease in IgAN. Our results suggest that certain interleukin-1 gene cluster variants (i.e., the T allele of rs16944 in *IL1B*) and haplotypes may be pathogenically involved in progressive IgAN. However, there exists a significant demand for multi-omics resources to further investigate the present findings and gain biological insights. Additionally, it is imperative to conduct future research involving functional experiments to confirm the significance of potential causal genetic variations of interleukin-1 gene cluster in the progressive IgAN.

## Figures and Tables

**Table 1:** Demographic and clinical characteristics of the participants in the case-control study on the risk of IgA nephropathy. *p* values provided for intergroup comparisons, i.e., between cases (progressive IgA nephropathy) and diseased controls (non-progressive IgA nephropathy), were calculated by the Mann–Whitney U test for continuous variables and by the χ^2^ test for categorical variables.

Parameters	Case-Control Study Population Groups (n = 367)				
	Healthy Controls	IgA Nephropathy *	Diseased Controls	Cases	*p*
n	246	121	54	67	n.a.
Sex (m/f)	182/64	88/33	34/20	54/13	0.030
Age (years)	44.6 ± 15.0	45.6 ± 15.2	42.3 ± 15.5	49.8 ± 14.3	0.012
IgA duration (years)	n.a.	5.9 ± 4.7	6.0 ± 4.9	5.7 ± 4.3	0.946
End-stage renal disease [n (%)]	-	15 (12.4)	0	15 (22.4)	<0.001
Mean blood pressure (mmHg)	-	72.7 ± 12.1	69.1 ± 10.1	75.3 ± 12.3	0.004
Macroscopic hematuria [n (%)]	-	19 (15.7)	11 (20.4)	8 (11.9)	0.205
Microscopic hematuria	-	85 (70.2)	34 (62.9)	51 (76.1)	0.099
Proteinuria (mg/d)	-	2170 ± 34,988	613 ± 471	3438 ± 4306	<0.001
Creatinine (mg/dL)	-	2.46 ± 3.01	1.07 ± 0.22	3.50 ± 3.65	<0.001
Hematocrit (%)	-	38.6 ± 7.1	41.6 ± 4.7	36.4 ± 7.7	<0.001

* All patients with histologically proven IgAN.

**Table 2:** Distribution of genotypes of *IL1* gene cluster among cases (progressive IgA nephropathy), disease (non-progressive IgA nephropathy), and healthy control subjects.

Gene Variant	Genotype	Progressive IgA Nephropathy	Diseased Controls	Healthy Controls	*p* Value	OR_G_ (95% CI)
			n (%)			
***IL1A C-899T*** ***rs1800587***	C C	30 (44.8)	30 (55.6)	120 (48.8)	0.458	0.97 (0.68–1.38)
	C T	30 (44.8)	22 (40.7)	98 (39.8)		
	T T	7 (10.4)	2 (3.7)	28 (11.4)		
***IL1B* C-511T** **rs16944**	C C	22 (32.8)	30 (55.6)	94 (38.2)	0.118	0.96 (0.67–1.35)
	C T	37 (55.2)	19 (35.2)	126 (51.2)		
	T T	8 (11.9)	5 (9.3)	26 (10.6)		
***IL1RN*** **allele L vs. 2**	L L	41 (61.2)	33 (61.7)	139 (56.5)	0.740	0.82 (0.57–1.19)
	L 2	23 (34.3)	17 (31.5)	84 (34.1)		
	2 2	3 (4.5)	4 (7.4)	23 (9.3)		

**Table 3:** Distribution of genotypes of *IL1* gene cluster among cases (progressive and non-progressive IgA nephropathy) and healthy control subjects.

Gene Variant	Genotype	IgA Nephropathy	Healthy Controls	*p* Value	OR_G_ (95% CI)
***IL1A C-899T*** ***rs1800587***	C C	60	120 (48.8)	0.482	0.91 (0.62–1.34)
	C T	52	98 (39.8)		
	T T	9	28 (11.4)		
***IL1B* C-511T** **rs16944**	C C	52	94 (38.2)	0.649	0.87 (0.59–1.28)
	C T	56	126 (51.2)		
	T T	13	26 (10.6)		
***IL1RN*** **allele L vs. 2**	L L	74	139 (56.5)	0.451	0.81 (0.54–1.21)
	L 2	40	84 (34.1)		
	2 2	7	23 (9.3)		

**Table 4:** Distribution of genotypes of *IL1* gene cluster among cases (progressive IgA nephropathy) and diseased control subjects (non-progressive IgA nephropathy).

Gene Variant	Genotype	Progressive IgA Nephropathy	Diseased Controls	*p* Value	OR_G_ (95% CI)
			n (%)		
***IL1A C-899T*** ***rs1800587***	C C	30 (44.8)	30 (55.6)	0.267	1.62 (0.83–3.15)
	C T	30 (44.8)	22 (40.7)		
	T T	7 (10.4)	2 (3.7)		
***IL1B* C-511T** **rs16944**	C C	22 (32.8)	30 (55.6)	0.041	2.11 (1.09–4.07)
	C T	37 (55.2)	19 (35.2)		
	T T	8 (11.9)	5 (9.3)		
***IL1RN*** **allele L vs. 2**	L L	41 (61.2)	33 (61.7)	0.772	0.95 (0.48–1.90)
	L 2	23 (34.3)	17 (31.5)		
	2 2	3 (4.5)	4 (7.4)		

**Table 5:** Results of linkage disequilibrium (LD) test between pairs of polymorphisms for cases with progressive IgAN and diseased controls with non-progressive IgAN (in brackets).

Progressive IgAN (Diseased Controls)		
	*IL1B*	*IL1RN*
*IL1A*	D’ = 0.438 (0.233)	D’ = 0.826 (0.131)
	r^2^ = 0.061 (0.047)	r^2^ = 0.118 (0.012)
	*p* = 0.004 (0.025)	*p* < 0.001 (*p* = 0.186)
*IL1B*		D’ = 0.497 (0.404)
		r^2^ = 0.111 (0.134)
		*p* < 0.001 (<0.001)

**Table 6:** Estimated haplotype frequencies for the three IL1 cluster gene polymorphisms (rs1800587, C-899T in *IL1A*; rs16944, C-511T in *IL1B*; and the 86 bp tandem repeat, VNTR *1–*5 in *IL1RN*) in patients with progressive IgAN and in patients with non-progressive IgAN.

Haplotypes	Haplotype Frequency			χ^2^ test	
(rs1800587, rs16944, 86 bp VNTR)	Progressive IgA Nephropathy	Diseased Controls	*p*-Value	OR (95% CI)	Overall *p*-Value
C C 1	0.282	0.454	0.005	0.456 (0.261~0.797)	
C C 2	0.049	0.097	0.155	0.485 (0.176~1.337)	
C T 1	0.141	0.133	0.814	1.094 (0.519~2.306)	0.003
C T 2	0.167	0.047	0.003	4.208 (1.545~11.50)	
T C 1	0.229	0.153	0.112	1.718 (0.877~3.363)	

**Table 7:** Study characteristics.

First Author, Year	Country	Racial Descent	IgA Nephropathy		Cases (n)	Controls (n)	HWE (*p*-Value)	Progression of IgA Nephropathy		Cases (n)	Controls (n)
			Selection Criteria and Demographic Data of Cases	Selection Criteria and Demographic Data of Healthy Controls				Selection Criteria and Demographic Data of Cases (Progressors)	Selection Criteria and Demographic Data of Controls (Non-Progressors)		
Liu 1997 [24]	China	Asians	Biopsy-proven IgA nephropathy (67 males, 30 females, ranging in age from 10 to 58 years).	Normal subjects (51 males and 47 females, ranging in age from 18 to 55 years), without renal diseases	97	98	-	-	-	-	-
Shu, 2000 [25]	China	Asians	Biopsy-proven IgA nephropathy (57 males, 54 females; mean age 30.3 years). Cases with Henoch–Schoenlein purpura not mentioned.	Healthy controls not matched to cases for age and gender; further demographic data not mentioned.	111	100	0.5	Increase in serum creatinine or more than 50% increase in daily proteinuria or appearance of hypertension.	Patients with stable renal disease or those in remission.	45	66
Syrjanen, 2002 [26]	Finland	Caucasians	Biopsy-proven IgA nephropathy (102 males, 65 females), no evidence of primary causes. Nine cases with Henoch–Schoenlein purpura.	Healthy blood donors (100 males, 100 females) from local center; not matched to cases for age and gender.	167	400	0.04	Presence of chronic renal failure (serum creatinine ≥ 125 μmol/L in males and ≥105 μmol/L in females) initially or rise of serum creatinine over 20% at follow-up.	Absence of chronic renal failure (serum creatinine ≥ 125 μmol/L in males and ≥105 μmol/L in females) initially or at follow-up.	26	140
Watanabe et al., 2002 [27]	Japan	Asians	Biopsy-proven IgA nephropathy	Individuals without a history of renal disease	106	74	-	-	-	-	-
Bantis, 2004 [28]	Germany	Caucasians	Biopsy-proven IgA nephropathy (93 males and 30 females with mean age at diagnosis of 37.1 ± 14 years)	44 males and 56 females without history of kidney diseases or arterial hypertension matched for age	123	100	-	Fast progressors	Low progressors	48	75
Chin 2005 [29]	Korea	Asians	Biopsy-proven IgA nephropathy	Normotensive individuals with no evidence of renal disease	108	100	-	-	-	-	-
Liu 2008 [30]	Canada	Caucasians	Biopsy-proven IgAN, exclusion of secondary IgAN	Healthy controls matched to cases for age and gender	255	187	>0.05				
Liu 2008 [30]	France	Caucasians	Biopsy-proven IgAN, exclusion of secondary IgAN	Healthy controls matched to cases for age and gender	271	205	>0.05				
Liu 2008 [30]	Finland	Caucasians	Biopsy-proven IgAN, exclusion of secondary IgAN	Healthy controls matched to cases for age and gender	206	111	>0.05				
Hahn 2009 [15]	Korea	Asians	Pediatric patients with biopsy-proven IgAN	Healthy controls	182	500	>0.05	-	-	-	-
Jung 2012 [31]	Korea	Asians	Biopsy-proven IgAN	Healthy controls	69	146	>0.05				
Yamamoto, 2012 [32]	Japan	Asians	Biopsy-proven IgA nephropathy patients aged between 25 and 50 years	Healthy hospital employees aged between 25 and 50 years.	230	262	0.80	-	-	-	-
Wang, 2013 [33]	China	Asians	Biopsy-proven primary IgAN with no evidence of systemic diseases such as diabetes, chronic liver disease, or systemic lupus erythematosus.	Gender- and age-matched healthy controls with no history of renal disease or hypertension.	527	543	0.45	-	-	-	-
Yang 2016 [34]	China	Asians	Biopsy-proven IgAN	Healthy controls	166	198	>0.05	-	-	-	-
Gao 2017 [35]	China	Asians	Biopsy-proven IgAN, exclusion of secondary IgAN	Healthy controls matched for age, gender, and ethnicity	351	310	>0.05	-	-	-	-
Zhang 2017 [36]	China	Asians	Biopsy-proven IgAN, exclusion of secondary IgAN	Healthy controls	417	463	>0.05	-	-	-	-

**Table 8:** Results regarding genotype counts.

GENE	VARIANT	RS	Studies (n)	Cases/Controls (n)	RE OR_G_	95% LL	95% UL	I^2^(%)/ Gamma	P_Q_/ SE(Gamma)	P_E_
*IL1A*	C-899T	rs1800587	2	594/789	0.94	0.71	1.24	0.00	0.75	-
*IL1B*	C-511T	rs16944	2	530/661	0.92	0.60	1.42	64.36	0.09	-
*IL1B*		rs1143627	1	178/495	1.44	1.08	1.93	0.18	0.07	-
*IL1RN*	allele L vs. 2		1	67/246	0.79	0.47	1.31	−0.12	0.13	-
*IL1RN*		rs928940	1	179/466	0.72	0.54	0.97	−0.16	0.07	-
*IL1RN*		rs439154	1	180/490	0.68	0.50	0.92	−0.19	0.07	-
*IL1RN*		rs315951	1	181/483	0.73	0.55	0.98	−0.15	0.07	-
*IL4R*		rs1805015	3	732/499	0.75	0.44	1.29	76.83	0.01	0.35
*IL5RA*		rs340833	3	729/503	1.12	0.61	2.06	19.20	<0.001	0.58
*IL6*		rs1800796	2	693/677	0.86	0.56	1.32	77.69	0.03	-
*IL10*	C-819Τ	rs1800871	2	581/572	0.79	0.64	0.97	0.82	0.37	-
*IL10*	-1082A>G	rs1800896	3	1108/1115	1.06	0.82	1.37	0.00	0.98	0.20
*IL18*	C-607A	rs1946518	3	465/606	0.81	0.46	1.45	85.89	<0.001	0.54
*IL18*	-137G/C	rs187238	2	298/408	1.05	0.74	1.47	0.00	0.74	-

**Table 9:** Results regarding allele counts.

GENE	VARIANT	RS	Studies (n)	Cases/Controls (n)	RE OR	95% LL	95% UL	P_Q_/ SE(Gamma)	P_E_
*IL1B*	-511C/T	rs16944	1	167/400	1.35	1.05	1.75	-	-
*IL1B*		rs1143627	1	417/463	1.21	1.00	1.46	-	-
*IL1RN*	Allele L vs. 2		4	481/672	1.56	1.00	2.44	0.127	0.19
*IL1RN*	Allele L vs. 2		1	67/246	0.79	0.47	1.31	0.12	0.13
*IL10*	C-819Τ	rs1800871	1	108/100	1.42	0.95	2.14	-	-
*IL10*	-1082A>G	rs1800896	2	123/100	0.61	0.23	1.60	0.043	-

## Data Availability

The datasets used and/or analyzed during the current study are available from the corresponding author on reasonable request.
